# Basic geriatric assessment does not predict in-hospital mortality after PEG placement

**DOI:** 10.1186/1471-2318-12-52

**Published:** 2012-09-06

**Authors:** Christine Smoliner, Dorothee Volkert, Anke Wittrich, Cornel C Sieber, Rainer Wirth

**Affiliations:** 1 Department of Internal Medicine and Geriatrics, St.-Marien-Hospital Borken, Am Boltenhof 7, 46325, Borken, Germany; 2 Institute for Biomedicine of Aging (IBA), Friedrich-Alexander-Universität Erlangen-Nürnberg, Nürnberg, Germany; 3 Bundesverband Geriatrie e.V, Reinickendorfer Str. 61, 13347, Berlin, Germany; 4 Department of Internal Medicine II, Nürnberg Hospital, Prof.-Ernst-Nathan-Str. 1, 90419, Nürnberg, Germany

**Keywords:** Percutaneous endoscopic gastrostomy (PEG), Mortality, Geriatric assessment

## Abstract

**Background:**

Percutaneous endoscopic gastrostomy (PEG) is an established procedure for long-term nutrition. However, studies have underlined the importance of proper patient selection as mortality has been shown to be relatively high in acute illness and certain patient groups, amongst others geriatric patients. Objective of the study was to gather information about geriatric patients receiving PEG and to identify risk factors associated with in-hospital mortality after PEG placement.

**Methods:**

All patients from the GEMIDAS database undergoing percutaneous endoscopic gastrostomy in acute geriatric wards from 2006 to 2010 were included in a retrospective database analysis. Data on age, gender, main diagnosis leading to hospital admission, death in hospital, care level, and legal incapacitation were extracted from the main database of the Geriatric Minimum Data Set. Self-care capacity was assessed by the Barthel index, and cognitive status was rated with the Mini Mental State Examination or subjectively judged by the clinician. Descriptive statistics and group comparisons were chosen according to data distribution and scale of measurement, logistic regression analysis was performed to examine influence of various factors on hospital mortality.

**Results:**

A total of 1232 patients (60.4% women) with a median age of 82 years (range 60 to 99 years) were included. The mean Barthel index at admission was 9.5 ± 14.0 points. Assessment of cognitive status was available in about half of the patients (n = 664), with 20% being mildly impaired and almost 70% being moderately to severely impaired. Stroke was the most common main diagnosis (55.2%). In-hospital mortality was 12.8%. In a logistic regression analysis, old age (odds ratio (OR) 1.030, 95% confidence interval (CI) 1.003-1.056), male sex (OR 1.741, 95% CI 1.216-2.493), and pneumonia (OR 2.641, 95% CI 1.457-4.792) or the diagnosis group ‘miscellaneous disease’ (OR 1.864, 95% CI 1.224-2.839) were identified as statistical risk factors for in–hospital death. Cognitive status did not have an influence on mortality (OR 0.447, CI 95% 0.248-1.650).

**Conclusion:**

In a nationwide geriatric database, no component of the basic geriatric assessment emerged as a significant risk factor for mortality after PEG placement, emphasizing individual decision-making.

## Background

Percutaneous endoscopic gastrostomy (PEG) is a common, safe and relatively economic procedure for long-term nutrition. However, some studies have underlined the importance of proper patient selection because mortality has been shown to be relatively high in acute illness and certain patient groups, amongst others geriatric patients. The 1-month mortality rates range from 6.5 to 23.9%, and 1-year mortality rates of more than 50% have been reported [1-4]. A recent British study observed that of those patients dying within 1-month after feeding tube placement, 40% died within the first week after the procedure [5]. The authors assumed that this mortality was most probably related to the acute disease and underlying co-morbidities and not to the procedure itself. Improper patient selection was held responsible for futile insertions and high morbidity [3,5-7]. This conclusion is supported by Janes et al., who reported a steeply increasing number of PEG placements in the last decade, resulting in higher mortality rates despite improved placement techniques and pointing towards a more liberal indication [8].

A variety of studies have tried to identify patient groups that may not benefit from PEG placement by analyzing differences in short-term mortality. Patient characteristics that have been shown to be associated with worse outcomes after feeding tube placement are: high age, increased number of comorbidities, hypoalbuminemia, elevated inflammatory markers and a low body mass index [3,9-13]. Most data on risk factors imply that disease severity may be responsible for high short term mortality. If this is true, one could expect functional limitations reflecting increased disease severity to indicate risk for PEG placement. Few studies have investigated the prognostic value of functional status to estimate the risk of poor outcome in geriatric patients with PEG placement [14,15].

The aim of the present study was to identify risk factors associated with in-hospital mortality in a large population of geriatric patients undergoing percutaneous endoscopic gastrostomy and to evaluate whether components of geriatric assessment could be useful for guiding decisions about PEG placement.

## Methods

### Study design and data collection

This observational study retrospectively analyzed data from the GEMIDAS project, a Geriatric Minimum Data Set, which serves as an instrument for voluntary quality assurance in acute and rehabilitative geriatric hospital units in Germany. The database includes information on age, gender, main and secondary diagnosis according to International Classification of Diseases (ICD) 10, length of stay, and the results of the basic geriatric assessment at hospital admission and discharge. Since 2004, operative and endoscopic procedures have been documented according to the German diagnostic related groups (G-DRG) [16], which allows the identification of patients with specific procedures, such as PEG placement.

The total number of patients included in the GEMIDAS database during the observation period between 2006 and 2010 was 199,454 from 68 hospitals all over Germany. Inpatients from 68 acute geriatric hospital units who had PEG-placement performed during the observation period were identified for this study. We received an anonymized data set of 1281 patients, which represented 0.64% of the GEMIDAS population. Forty-nine patients had to be excluded from the analysis due to double entry or incomplete data regarding gender, age, main diagnosis, length of stay, or mortality.

The following variables were extracted from the main database: age, gender, main diagnosis according to ICD-10, length of stay, place that person was discharged to or death in hospital, care level and legal incapacitation, Barthel index, and cognitive status. The main diagnoses captured by ICD-10 codes were pooled for diagnosis groups. In the G-DRG, the main diagnosis is based on the reason leading to hospital admission. In Germany, a care level is allocated to a person in need of care; depending on the severity, a level from 1 to 3 is assigned. Care level 1 is attributed to a person who needs care in some areas, adding up to a minimum of 90 minutes per day. Care level 3 is attributed to persons who are completely unable to care for themselves and given up to 5 hours of care. In our population, several patients did not have a care level upon admission despite a low Barthel index, which reflects the acute situation of the geriatric patient, who could have been completely independent before admission to the hospital. The ability to perform basic activities of daily living (ADL) was assessed by the Barthel index at admission and discharge, with a range from 0 to 100 points in which 0 points represented complete care dependency [17]. The ability to walk was extracted from the corresponding component of the Barthel index and included patients who were able to walk with amateur help, a walking frame, or were completely independent. Cognitive status was rated with the Mini Mental State Examination (MMSE) [18]. A score of 0 to 19 points was considered to represent moderate or severe cognitive impairment, 20 to 25 indicated mild or questionable cognitive impairment, and more than 25 points indicated normal cognitive status [19]. If the MMSE score was not available, as was the case in several patients e.g. due to severe sickness, aphasia after stroke, etc. cognitive status was evaluated by the attending physician. On the basis of the subjective clinical judgment patients were categorized into three groups: no cognitive impairment, slight or questionable cognitive impairment, and moderate to severe cognitive impairment. As this was a retrospective database analysis with an anonymized dataset, an ethics approval was not necessary.

### Statistical analysis

Statistical analyses were performed using PAWS Version 18 (IBM SPSS Statistics, Chicago, USA). Descriptive statistics were used for patients' baseline characteristics. A comparison of means was performed using the *t*-test or Mann–Whitney-*U* test according to the data distribution. The chi-square test was applied to detect differences between nominal data. In order to examine the influence of the factors age, gender, main diagnosis, Barthel index, and cognition on hospital mortality, a binary logistic regression analysis was performed. The level of significance was determined *a priori* at p < 0.05.

## Results

A total of 1232 patients with a median age of 82 (range 60-99) years were included in the analysis. The patients’ baseline characteristics are shown in Table 1. Approximately two-thirds of the patients were women. Even though more than 40% of the patients did not have a care level at admission, Barthel index values at admission and discharge were very low, which shows how severely sick included patients were. Only 4.7% of the patients were able to walk at admission. Information on cognition was available for 664 patients (376 patients assessed with MMSE, 288 patients assessed by the attending doctor), of which 20.2% showed signs of minor cognitive impairment and 67.8% were moderately to severely impaired.

**Table 1:** Baseline characteristics of study participants (n = 1232)

**Characteristic**	**n (%) or Mean ± SD**
Gender, female	744 (60.4)
Age, years	81.6 ± 6.9
Main Diagnosis	
Malnutrition	41 (3.3)
Miscellaneous	235 (19.1)
Musculoskeletal disease	102 (8.3)
Stroke	680 (55.2)
Other diseases potentially accompanied by dysphagia	98 (8.0)
Pneumonia	76 (6.2)
Length of hospital stay, days	26.1 ± 12.3
Barthel index at admission (n = 1215)	9.5 ± 14.0
Barthel index at discharge (n=1082)	12.0 ± 16.2
Care Level (n = 1169)	
No care level	499 (42.7)
1(low)	277 (23.7)
2(medium)	287 (24.6)
3(high)	106 (9.1)
Walking at admission (n = 1125)	
Able to walk	53 (4.7)
Not able to walk	1072 (95.3)
Walking at discharge based on Barthel index (n = 1087)	
Able to walk	87 (8.0)
Not able to walk	1000 (92.0)
Mini Mental State Exam score (n = 376)	14.2 ± 9.5
Cognitive rating (n = 664)*	
No cognitive impairment	80 (12.0)
Mild or questionable cognitive impairment	134 (20.2)
Moderate to severe cognitive impairment	450 (67.8)
Legal Incapacitation (n = 1087)	
No legal incapacitation	495 (45.9)
Legal incapacitation	583 (54.1)
No information	154 (12.5)
Discharged to (n = 1216)	
Private household	355 (29.2)
Nursing home	504 (41.4)
Death in hospital	156 (12.8)
Other	201 (16.5)

^*^ Patients assessed with MMSE and/or evaluation by attending physician.

Stroke was the most common diagnosis leading to hospital admission in these patients, affecting more than half of the study population. The second largest group was 'miscellaneous', which included cancer, epilepsy, heart failure and infection, as well as other diseases such as those of the skin and the gastrointestinal tract. The diagnosis group ‘malnutrition’ included synonyms for reduced nutritional status, including protein-energy malnutrition, cachexia, and dehydration. Diseases other than stroke that are potentially accompanied by dysphagia were put in one group including Parkinson’s disease, dementia (26 patients, 2.1% of the study population), dysphagia of unknown cause and motoneuron disease. The diagnosis group pneumonia also included aspiration pneumonia (2,8%). Musculoskeletal diseases included fractures, osteoporosis with fractures, musculoskeletal disorders with surgery, osteoarthritis, and abnormalities of gait and mobility.

A total of 156 patients (12.8%) died over the course of their hospital stay, with a median survival of 20 days (range 1-81 days, interquartile 13 - 28) in those patients. In a group comparison of patients who survived and those who died in the hospital, patients with pneumonia or a miscellaneous diagnosis had significantly higher mortality rates that patients from other diagnosis groups. Interestingly, significantly more men than women died. Cognitive status at hospital admission was not significantly different in patients who survived and those who died (Table 2).

**Table 2:** Characteristics of survivors and patients who died in hospital

	**Survived hospital stay**	**Death in hospital**	**p-Value**
	**n = 1076**	**n = 156**	
Men	409 (33.2)	79 (6.4)	0.003
Women	667 (54.1)	77 (6.2)	
Age, years	81.5 ± 6.8	82.3 ± 7.9	n.s.
Diagnosis Groups			<0.0001
Malnutrition	38 (3.1)	3 (0.2)	
Miscellaneous	192 (15.4)	44 (3.6)	
Musculoskeletal disease	92 (7.5)	10 (0.8)	
Stroke	605 (49.1)	75 (6.1)	
Other diseases potentially accompanied by dysphagia	93 (7.5)	5 (0.4)	
Pneumonia	57 (4.6)	19 (1.5)	
Length of stay, days	26.6 ± 11.9	22.9 ± 13.9	0.001
Barthel index at admission	9.5 ± 13.9	9.8 ± 14.3	n.s.
No care level	440 (37.6)	59 (5.0)	n.s.
Care level 1	244 (20.9)	33 (2.8)	
Care level 2	245 (21.0)	42 (3.6)	
Care level 3	94 (8.0)	12 (1.0)	
Able to walk at admission	49 (4.4)	4 (0.4)	n.s.
Not able to walk at admission	928 (82.5)	144 (13.4)	
No cognitive impairment	74 (6.0)	6 (0.5)	n.s.
Mild or questionable cognitive impairment	122 (9.9)	12 (1.0)	
Moderate or severe cognitive impairment	395 (32.1)	55 (4.5)	
No legal incapacitation	427 (39.6)	68 (6.3)	n.s.
Legal incapacitation	512 (47.5)	71 (6.6)	

Data are given as n (%) or mean ± SD, *n.s*. = not significant.

In a regression analysis, risk factors for in-hospital mortality were old age, male sex, and the diagnosis groups miscellaneous or pneumonia (Table 3). Because cognitive status was available in only about half of the patients, we calculated a separate logistic regression model that included age, gender, main diagnosis, Barthel index, and cognitive status. In this model, the cognitive rating did not have an influence (odds ratio (OR) 0.447, 95% confidence interval (CI) 0.248-1.650). Age was not a significant risk factor anymore, whereas gender and diagnosis groups miscellaneous and pneumonia remained significant in the model. Interestingly, even though male sex was a risk factor for increased mortality in the regression analysis, women were significantly older (79.5 ± 7.1 vs. 83.1 ± 6.5 years; p = 0.016), and the Barthel index values were significantly lower (10.5 ± 15.3 vs. 8.9 ± 13.0; p = 0.011) and legal incapacitation was more prevalent (49.7% vs. 57.0%; p = 0.018) among women, indicating that women were more dependent overall (data not shown). Diagnosis groups did not differ between men and women.

**Table 3:** Binary logistic regression analysis with In-hospital mortality as the dependent variable

**Covariate**	**Estimated coefficient**	**Standard error**	**Odds ratio**	**95% Confidence interval**	**p-Value**
Constant term	-4.738	1.119			<0.0001
Old age	0.029	0.013	1.030	1.003-1.056	0.026
Male sex	0.555	0.183	1.741	1.216-2.493	0.002
Diagnosis group (vs. stroke)					
1 Malnutrition	-0.783	0.738	0.457	0.108-1.939	n.s.
2 Miscellaneous	0.623	0.215	1.864	1.224-2.839	0.004
3 Musculoskeletal	-0.116	0.361	0.891	0.439-1.807	n.s.
4 Diseases potentially accompanied by dysphagia	-0.755	0.478	0.470	0.184-1.200	n.s.
5 Pneumonia	0.972	0.304	2.641	1.457-4.792	0.001
Barthel index at admission	-0.000	0.006	1.000	0.988-1.012	n.s.

*n.s*. = not significant.

## Discussion

The present study found that old age, male gender and certain diagnoses were associated with higher in-hospital mortality after PEG placement in a geriatric population. The data shows no influence of the functional components of a basic geriatric assessment on mortality.

Hospital mortality in the described patient population was 12.8%, which is within the range of mortality rates observed in other studies of PEG-placement in geriatric patients [8,10].

Our finding that old age is predictive of decreased survival is supported by several studies in elderly patients conducted with different time frames for follow-up ranging from 1 to 36 months [1,3,11,20]. However, the increased risk was very small in our study; thus, age *per se* should have no impact on clinical decision making regarding whether a feeding tube should be placed or not.

The increased risk for in-hospital mortality observed in men is noteworthy and has been shown in other studies of the elderly [1,4,9,21]. Women in our population were generally more dependent, but diagnosis groups did not seem to differ between sexes. One possible explanation could be a more generous prescription of feeding tubes for men because they were significantly younger and had a higher functional status and lower care level, which possibly made them seem more promising as candidates with benefit from PEG placement. Even though we did not detect any gender differences in regards to the main diagnosis, men could have been sicker overall. Also, men comprised 40% of the present study population, but they account for 30% of the general GEMIDAS (years 2002 to 2008) population, supporting the previously mentioned hypothesis of a more generous prescription in men. We think this should not lead to any impact on decision-making, but it is an interesting finding prompting further research.

While patients in the pneumonia and miscellaneous groups had a statistically increased risk for in-hospital mortality it remains important to carefully evaluate indication and timing for PEG placement in every patient. Taking into account the prognosis and usefulness of the intervention in acutely ill patients have to be underlined. This is supported by studies that found patients with acute illness to be at a high risk of serious adverse events after PEG insertion [6,7]. Abuksis et al. suggested for instance the use of a nasogastric feeding tube during the first 30 days and then deciding on PEG insertion [6].

PEG placement in patients with a very low Barthel index and high cognitive impairment as is often the case in patients with advanced dementia is not supported by the literature; whether patients profit in terms of survival and improved nutritional or functional status or quality of life is questionable [10,22]. In our analysis, the Barthel index was not predictive of in-hospital mortality. However, the mean Barthel index value was extremely low, which could be the reason for not being able to detect an influence. The index values of patients in this study were similar to those of patients receiving PEG in an earlier study using GEMIDAS data (9.5 ± 13.0 vs. 8.2 ± 14.6), whereas the mean Barthel index value of the general GEMIDAS population is significantly higher (44.6 ± 26.8) [23]. Other studies investigating PEG placement in geriatric patients found very low functional status in patients receiving PEG feeding [14,15]. In our study, impaired functional status was not associated with increased in-hospital mortality. Thus, not functional status *per se*, but diagnosis and general prognosis should support the decision-making process.

Several studies and meta-analyses have emerged that question the practice of feeding tube placement in patients with dementia [22,24] Mitchell et al. for instance observed nursing home residents with advanced dementia and found that 40.7% of residents underwent burdensome interventions including aggressive nutrition therapy in the last 3 months of life [25]. Sanders et al. on the other hand compared the 30-day mortality after PEG placement in patients with dementia to other patient subgroups and found a significantly higher mortality in cognitively impaired patients (54% mortality vs. 28% mortality entire cohort, p <0,0001) [26]. In our study we were not able to find an influence of cognitive status on short-term survival in regards to hospital mortality. However, this conclusion is of limited value as cognitive status has only been available in approximately half of the patients and the origin of cognitive dysfunction (dementia, delirium, or other) could not be traced back. Taken together, our data indicate that cognitive impairment as a functional measure does not increase the risk of short-term mortality after PEG insertion.

The limitations of this study are the retrospective nature of the database analysis and the lack of information concerning several known risk factors such as for example secondary diagnoses, inflammatory markers, etc. that have been found to be significant predictors of decreased survival in patients with PEG placement [27].

Mini Mental Status Examination is used for cognition screening in the GEMIDAS-database. It has to be mentioned that the test is limited in differentiating between mild to no dysfunction of cognitive status and is of limited use in inpatients with dysphasia. It remains to discuss whether another instrument for more precise cognition screening should be used in the future.

Certainly it has to be mentioned that advanced directives play a certain role in patient selection, this however, could not be displayed by our study.

## Conclusion

Recent studies have reported high rates of early mortality after PEG placement in geriatric patients. In these particular cases, PEG placement might be regarded as futile or possibly introducing additional morbidity, evoking the question of whether early mortality and by this futile insertions can be partly predicted by certain patient characteristics or the geriatric assessment. In this context no component of the basic geriatric assessment emerged as a significant risk factor for short-term mortality after percutaneous endoscopic gastrostomy.

## Competing interests

There is no competing interest. This work has not received any financial support.

## Authors’ contributions

CS and RW performed data analysis, data interpretation and writing of the manuscript. AW performed data extraction from the GEMIDAS-database, DV, CCS and AW critically reviewed and edited the manuscript. All authors read and approved the final manuscript.

## Pre-publication history

The pre-publication history for this paper can be accessed here:

http://www.biomedcentral.com/1471-2318/12/52/prepub
